# Accelerated tumor growth under intermittent hypoxia is associated with hypoxia-inducible factor-1-dependent adaptive responses to hypoxia

**DOI:** 10.18632/oncotarget.18644

**Published:** 2017-06-27

**Authors:** Dae Wui Yoon, Daeho So, Sra Min, Jiyoung Kim, Mingyu Lee, Roza Khalmuratova, Chung-Hyun Cho, Jong-Wan Park, Hyun-Woo Shin

**Affiliations:** ^1^ Obstructive Upper Airway Research (OUaR) Laboratory, Department of Pharmacology, Seoul National University College of Medicine, Seoul 03080, Korea; ^2^ Department of Biomedical Science, Seoul National University Graduate School, Seoul 03080, Korea; ^3^ Ischemic/Hypoxic Disease Institute, Seoul National University College of Medicine, Seoul 03080, Korea; ^4^ Cancer Research Institute, Seoul National University College of Medicine, Seoul 03080, Korea; ^5^ Department of Otorhinolaryngology-Head and Neck Surgery, Seoul National University Hospital, Seoul 03080, Korea

**Keywords:** obstructive sleep apnea, cancer, hypoxia-inducible factor, intermittent hypoxia, hypoxia adaptation

## Abstract

Mounting evidence has revealed a causative role of intermittent hypoxia (IH) in cancer progression in mouse models of obstructive sleep apnea (OSA), but most studies have focused on the effects of IH following tumor implantation using an exposure to single IH frequency. Thus, we aimed to investigate 1) the potential effect of IH on the initial tumor growth in patients with OSA without an interaction with other mechanisms induced by IH in mice and 2) the influence of the IH frequency on tumor growth, which were tested using pre-conditioning with IH (Pre-IH) and 2 different IH frequencies, respectively. Pre-IH was achieved by alternatively maintaining melanoma cells between normoxia (10 min, 21% O_2_) and hypoxia (50 min, 1% O_2_) for 7 days (12 cycles per day) before administering them to mice. The conditions for IH-1 and IH-2 were 90 s of 12% FiO_2_ followed by 270s of 21% FiO_2_ (10 cycles/h), and 90 s of 12% FiO_2_ and 90 s of 21% FiO_2_ (20 cycles/h), respectively, for 8 h per day. Tumor growth was significantly higher in the Pre-IH group than in the normoxia group. In addition, the IH-2 group showed more accelerated tumor growth compared to the normoxia and IH-1 groups. Immunohistochemistry and gene-expression results consistently showed the up-regulation of molecules associated with HIF-1α-dependent hypoxic adaptation in tumors of the Pre-IH and IH-2 groups. Our findings reveal that IH increased tumor progression in a frequency-dependent manner, regardless of whether it was introduced before or after *in vivo* tumor cell implantation.

## INTRODUCTION

Obstructive sleep apnea (OSA) is a common nocturnal respiratory symptom affecting 2% to 5% of adults in the general population [1, 2]. Patients with OSA undergo repetitive partial or complete obstruction of the upper airway during sleep. OSA results in frequent sleep-fragmentation events, intrathoracic negative-pressure swings, and intermittent hypoxia (IH). Those characteristics are regarded as potential pathophysiological factors for the development or worsening of several chronic diseases associated with OSA, including cardiovascular diseases, metabolic diseases, and neuropsychiatric problems.

The severity of OSA is typically determined by the number of hypopnea (partial obstruction) and apnea (complete obstruction) events per h of sleep, referred to as the apnea–hypopnea index (AHI) and categorized as mild (5 ≤ AHI < 15), moderate (15 ≤ AHI < 30), or severe (30 ≥ AHI). Numerous previous findings have shown that the risk or severity of chronic diseases and pathological conditions are dose-dependently associated with the OSA severity [3–6].

Several recent epidemiological and experimental findings have shown a significant association between OSA and cancer risk. The Wisconsin Sleep Cohort Study was performed to examine the association between OSA and cancer-specific mortality [7]. The results confirmed that significantly higher hazard ratios (HRs) for cancer mortality occurred in the severe-OSA group (HR, 4.8; 95% CI, 1.7–13.2) compared to non-OSA subjects, with a trend toward increasing cancer mortality according to OSA severity. In addition, the results of a Spanish multicenter study showed a significant relationship between OSA and cancer incidence [8]. Interestingly, it appears that specific cancer types are particularly associated with OSA. According to a study performed to analyze the health insurance data of 5.6 million individuals, the risks for pancreatic cancer, kidney cancer, and melanoma were significantly higher in OSA patients [9], while the risks for breast, colorectal, and prostate cancer were lower in OSA patients than in a control group. Although these epidemiological studies revealed a positive association between OSA and cancer, several confounding variables (e.g., age, obesity, and lifestyle) make it difficult to clarify the direct causal effect of OSA on cancer risk.

It has been well documented that hypoxia frequently occurs in solid tumors. This is because the density of the blood vessels is not sufficient to support the perfusion of whole tumors, which rapidly proliferate. Thus, some tumor cells receive an insufficient oxygen supply. To overcome or adapt to hypoxic conditions resulting from the lack of oxygen supply, tumors promote formation of their own vascular network via hypoxia-inducible factor-1 (HIF-1), a master regulator of expression of several genes under hypoxia (e.g., angiogenesis, metabolism, metastasis, or glucose uptake) [10, 11]. Two types of hypoxia occur within tumor tissues, namely chronic hypoxia and IH. Accumulating evidences consistently reported that the tumor interior is close to IH conditions with spatial and temporal changes in hypoxia, as evidenced by dynamic IH measurements [12] and indirect measurements of fluctuating tumor blood flow [13]. It has been reported that HIF-1 is more accumulated in cells exposed to IH than those undergoing chronic hypoxia [14, 15]. It is noteworthy that HIF-1 has been regarded as a therapeutic target for cancer progression, and the level of vascular endothelial growth factor (VEGF), which is a downstream of HIF-1 correlated with tumor weights in a murine model of OSA [16].

**Figure 1:**
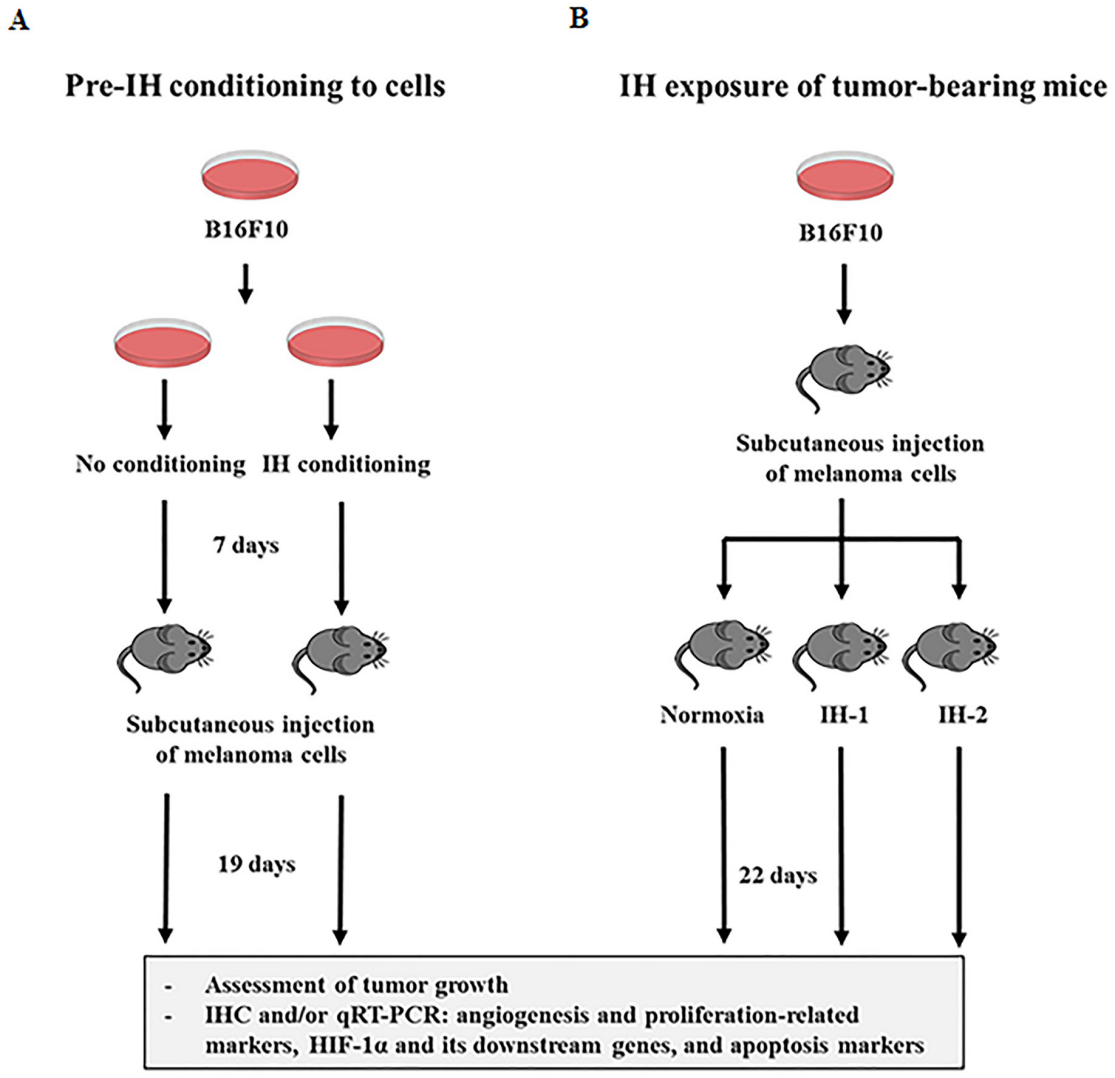
Schematic representation of the experimental protocols used in this study **(A)** Mouse melanoma B16F10 cells were preconditioned by intermittent hypoxia (Pre-IH) with alternating cycles of 1% O_2_ for 50 min, or 21% O_2_ for 10 min (12 cycles/day for 84 total cycles), or maintained under normoxia (N; no conditioning) for 7 days before subcutaneous injection into C57BL/6 mice. Then, the tumor-bearing mice were maintained in ambient room air for 19 days without additional exposure to intermittent hypoxia (IH). **(B)** B16F10 cells maintained under normoxia were subcutaneously injected into C57BL/6 mice, and then the mice were randomly divided into the N, IH-1, and IH-2 groups. Mice in these groups were then subjected to normoxia, IH-1 (90 s of 12% FiO_2_ followed by 270s of 21% FiO_2_ [10 cycles/h]), or IH-2 (90 s of 12% FIO_2_ and 90 s of 21% FIO_2_ [20 cycles/h]) conditions for 22 days. Tumor growth was assessed by measuring estimated tumor volumes during the experimental period. Tumors were excised from the mice and processed for immunohistochemical staining and quantitative real-time polymerase chain reaction analysis.

IH conditions mimicking O_2_-saturation profiles with recurrent apneic events seen in patients with OSA, promoted tumor progression and aggressiveness in mouse xenograft models [17–19]. However, these studies mostly used experimental protocols wherein mice were exposed to IH following tumor cell injection and were then monitored for longitudinal tumor changes; thus, only the effect of IH on tumor progression could be explored. Moreover, despite indications that the severity of OSA is involved in the worsening or development of morbidities associated with OSA, most studies have used only a single IH frequency in the study design. Thus, these studies did not elucidate whether tumor growth or malignancy were influenced by the frequency of IH. With this in mind, we established 2 different experimental protocols. First, tumor cells were preconditioned by IH prior to injection into mice without additional IH exposure during *in vivo* tumor progression (Figure 1A). This protocol may explain the potential effect of IH on the initial tumor growth in patients with OSA, without an interaction with other mechanisms induced by IH in mice. Second, tumor cells maintained under normoxia were injected into mice, and then 2 different protocols involving either 10/h or 20/h of continuous IH were used with the mice during tumor progression (Figure 1B). The 2 different IH conditions of 10/h and 20/h represent mild and moderate OSA, respectively. Melanoma cells were selected for this study because a higher risk of melanoma has been reported in patients with OSA [9].

## RESULTS

### Pre-intermittent hypoxia (Pre-IH) conditioning promoted *in vivo* tumor growth

**Figure 2:**
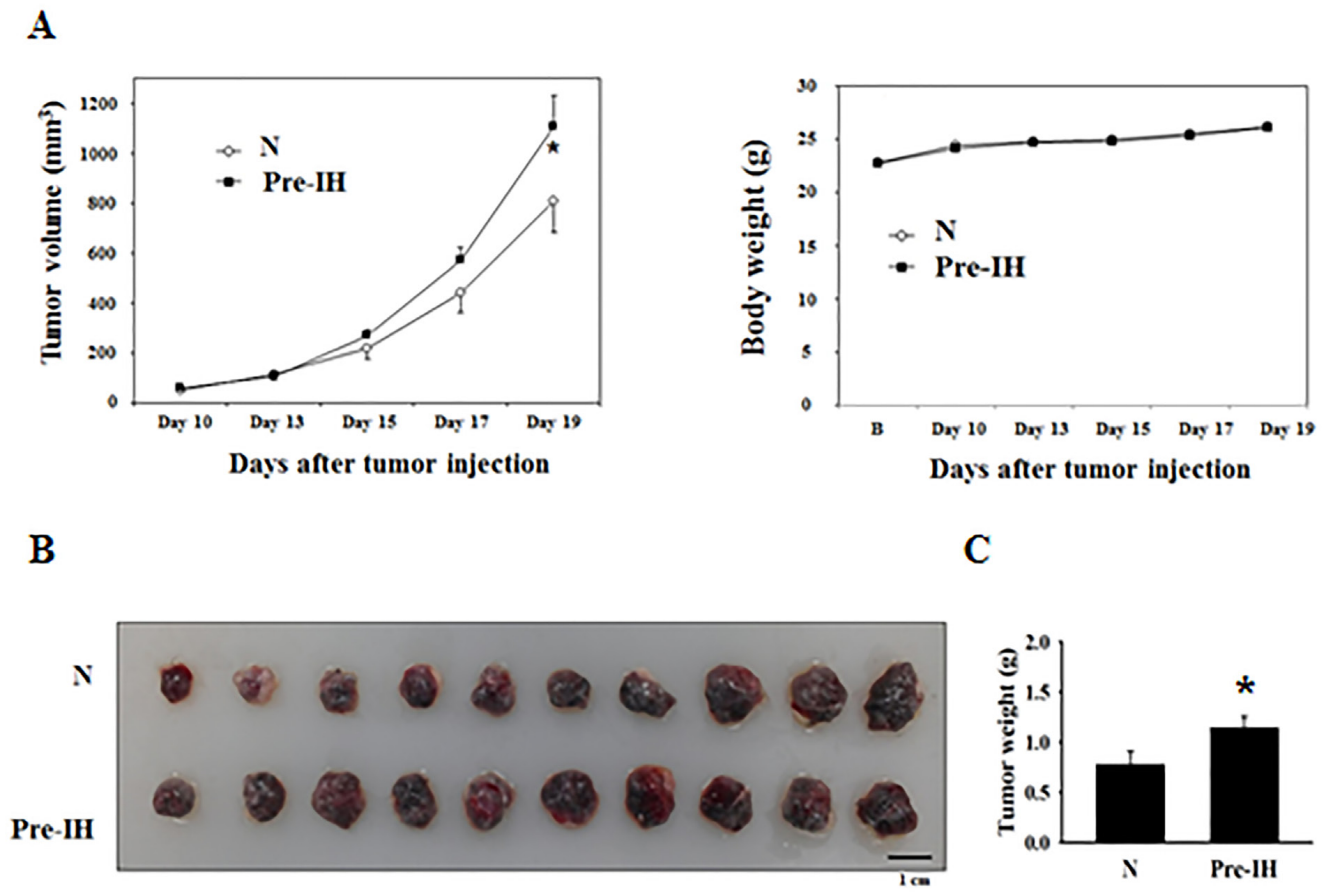
Tumor growth in mice in the no-conditioning (N) and pre-intermittent, hypoxia-conditioning (Pre-IH) groups **(A)** Changes in tumor volumes and body weights. Tumor volumes were measured every 2 or 3 days starting from day 10 post-tumor injection. At day 19, tumor volumes in mice in the Pre-IH group were significantly higher than those in the N group (n = 10/group). **(B)** Comparison of tumor sizes from mice in the N and Pre-IH groups. **(C)** Comparison of tumor weights in the N and Pre-IH groups. Data are presented as the mean ± S.E.M. ^*^P < 0.05 compared to N group, as determined by the Mann–Whitney U test.

Preconditioning of melanoma cells by IH was achieved by alternatively maintaining melanoma cells between normoxia (10 min, 21% O_2_) and hypoxia (50 min, 1% O_2_) for 7 days (12 cycles per day). Non-conditioned cells were continuously maintained in the normoxic chamber. Mice inoculated with Pre-IH-conditioned melanoma cells exhibited more accelerated tumor growth than those of the no-conditioning (N) group did at day 19 post-tumor injection (left panel in Figure 2A and 2B). Tumor weights were also significantly higher in the Pre-IH group than in the N group (Figure 2C). No significant difference in body weight was observed between the 2 groups throughout the experimental period (right panel in Figure 2A).

We examined whether Pre-IH conditioning would induce alterations in HIF-1α, HIF-2α, or pyruvate dehydrogenase kinase 1 (PDK1; a downstream effector of HIF) expression during the adaptation of cells to hypoxia (Supplementary Figure 1A). Cells exposed to 7 days of N or Pre-IH conditioning were lysed and used for western blot analysis. HIF-1α was rarely expressed in both N and Pre-IH cells. HIF-2α expression was slightly higher in the Pre-IH group than in the N group. Pre-IH-conditioned cells showed higher PDK1 expression than did the N cells, indicating an accumulated hypoxic burden of Pre-IH-conditioned cells. Prior to performing cell cycle analysis and proliferation assays, cells grown under N or Pre-IH conditions for 7 days were further incubated overnight under normoxic conditions. The proportion of DNA at different cell cycle stages (G1/G0, S, and G2/M) was assessed by flow cytometry in N- and Pre-IH-conditioned cells. As shown in Supplementary Figure 1D, Pre-IH conditioning did not affect any cell cycle stage in B16F10 cells. In BrdU-based cell-proliferation assays, no difference was observed in proliferation between N- and Pre-IH-conditioned cells (Supplementary Figure 1E). These results suggested that 7 days of Pre-IH conditioning did not alter the proliferative potential of tumor cells *in vitro*.

### Pre-IH conditioning increased HIF-1α-dependent adaptive responses to hypoxia in tumors of xenografted mice

**Figure 3:**
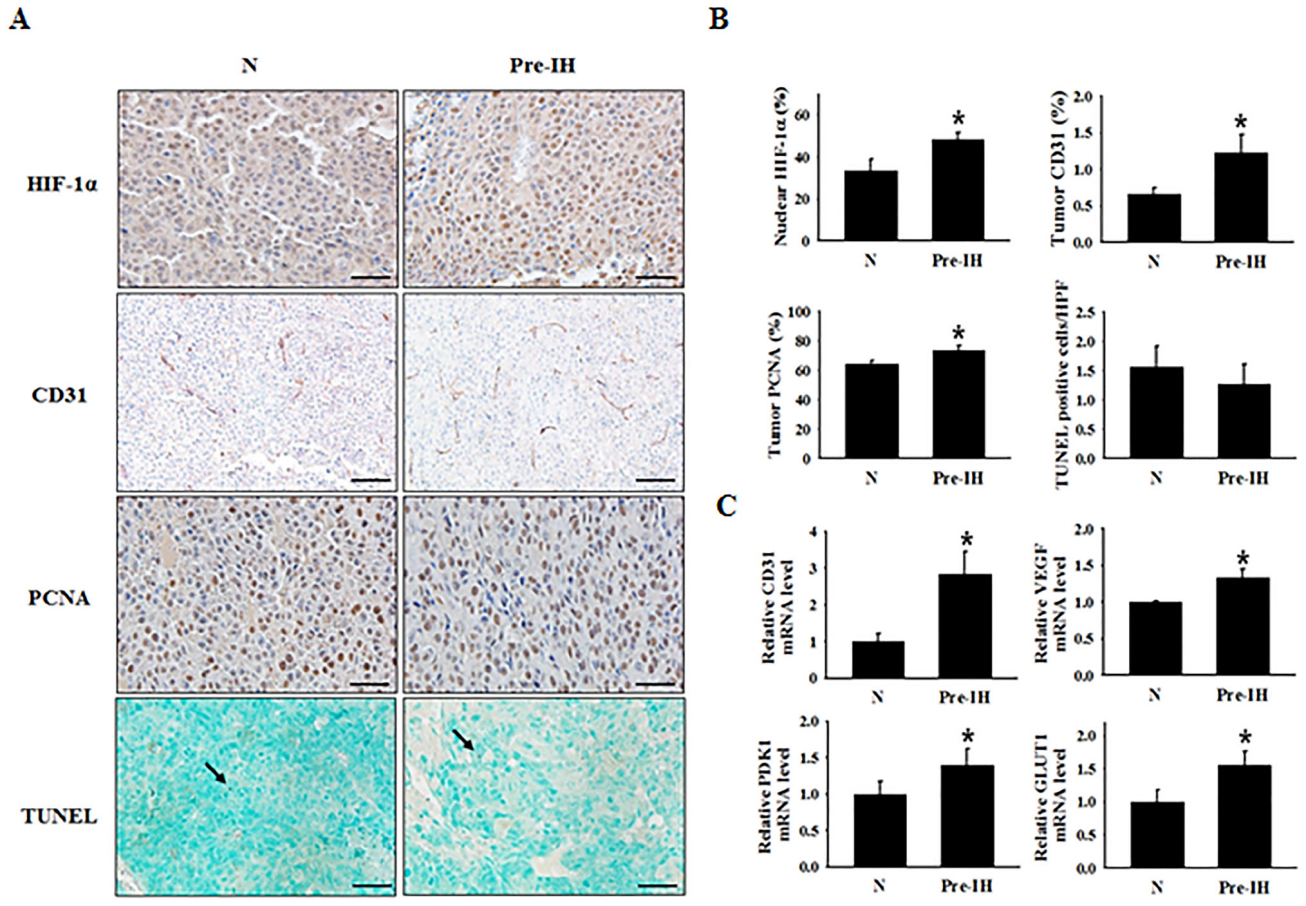
Expression of a cell-proliferation marker, endothelial cell markers, and hypoxia-response transcription factor in tumor tissues from mice in the no-conditioning (N) and pre-intermittent hypoxia conditioning (Pre-IH) groups **(A)** Representative immunohistochemical staining images for HIF-1α, PCNA, CD31, and TUNEL-positive nuclei. Tumor tissues were collected on day 19 post-tumor injection. **(B)** Quantification of the % of cells staining positive for nuclear HIF-1α, the % CD31-stained area, the % PCNA-positive cells, and the average number of TUNEL-positive cells per high-power field. **(C)** Relative *CD31*, *VEGFA*, *PDK1*, and *GLUT1* mRNA-expression levels in tumors from mice in the N and Pre-IH groups. The arrow indicates TUNEL-positive nuclei. Scale bars, 50 μm except for CD31 (100 μm). ^*^P < 0.05 compared to the N group, as determined by the Mann–Whitney U test.

We performed immunohistochemical staining (IHC) to examine whether Pre-IH conditioning affects adaptive responses to hypoxia in mouse tumors. Nuclear HIF-1α expression was significantly higher in tumors from mice inoculated with Pre-IH-conditioned cells than in tumors from mice inoculated with N-conditioned cells (Figure 3A and 3B). Given that HIF-1α is a well-known transcription factor for VEGF, which stimulates angiogenesis, we examined whether angiogenesis was increased in tumors of the Pre-IH group. Expression of cluster of differentiation 31 (CD31), an endothelial cell and angiogenesis marker, was significantly up-regulated in tumors in the Pre-IH group compared to those in the N group. Proliferation of tumor cells also increased in tumors of the Pre-IH group, as demonstrated by up-regulation of the proliferating cell nuclear antigen (PCNA). There was no difference in the number of terminal deoxynucleotidyl transferase dUTP nick end labeling (TUNEL)-positive nuclei between the 2 groups, implying that accelerated tumor growth in the Pre-IH group was not due to increased cell survival, but was due to the promotion of cell proliferation. To confirm the up-regulated expression of HIF-1α in IHC, we performed quantitative real-time polymerase chain reaction (qRT-PCR) and examined the expression of HIF-1α (Supplementary Figure 1A) and its downstream target genes. As shown in Figure 3C, expression of HIF-1α downstream target genes such as *VEGF-A*, glucose transporter 1 (*GLUT1)*, and *PDK1* increased in tumors of the Pre-IH group, compared to those of the N group. However, no significant difference was observed in the expression level of HIF-1α itself between the Pre-IH and N groups (Supplementary Figure 1B).

### Post-IH conditioning increased *in vivo* tumor growth in a frequency-dependent manner

**Figure 4:**
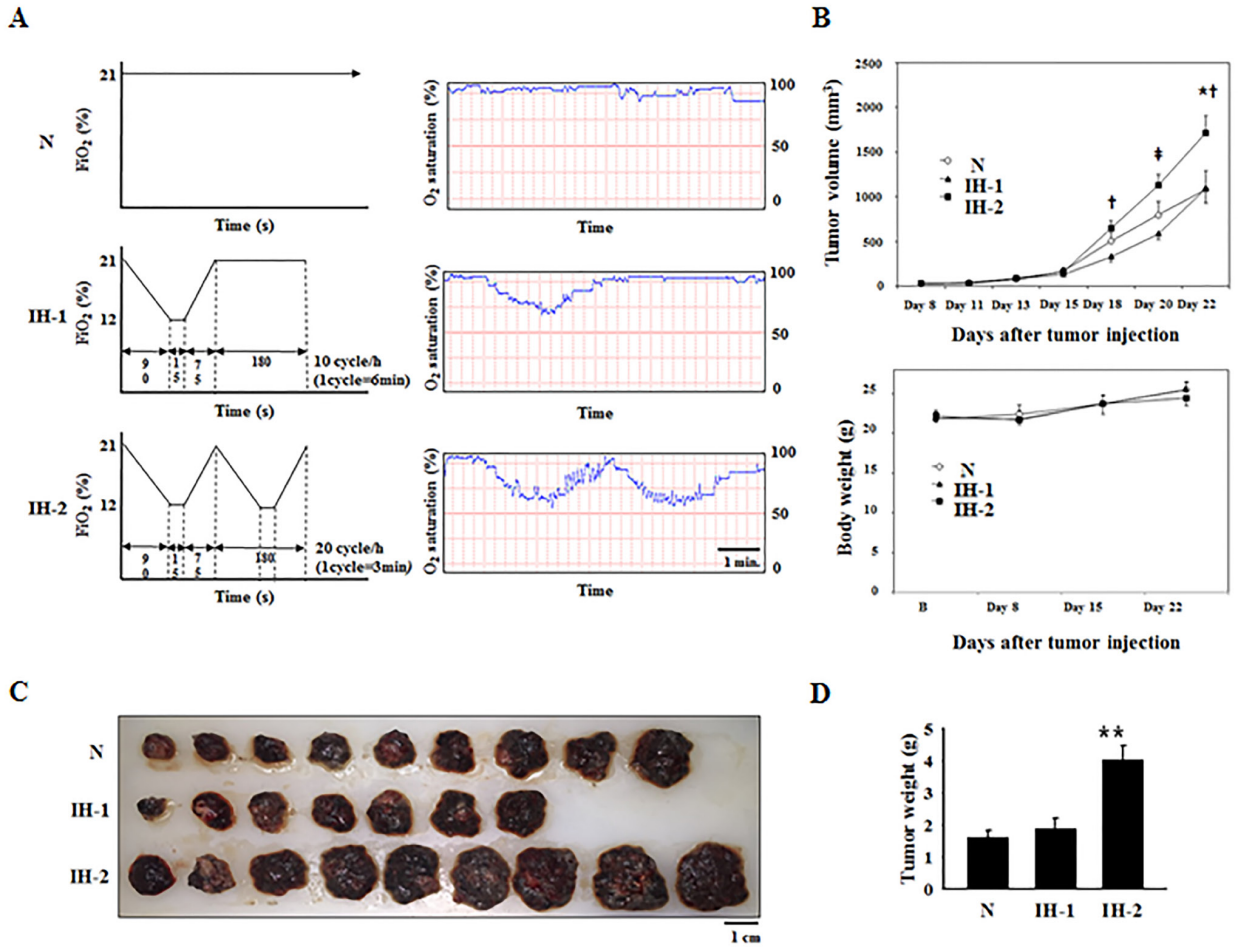
Tumor growth in mice subjected to normoxia (N), intermittent hypoxia-1 (IH-1), or intermittent hypoxia-2 (IH-2) (n = 10/group initially) **(A)** Time course of oxygen-saturation profiles measured by mouse oximetry in mice during exposure to N, IH-1, or IH-2 conditions. Mice in the N group were maintained under a constant FiO_2_ of 21%. The experimental parameters used for 1 cycle of IH-1 were 21%→12% FiO_2_ for 90 s, 12% FiO_2_ for 15 s, 12%→21% FiO_2_ for 75 s, and 21% FiO_2_ for 180 s (10 cycles/h). The parameters for 1 cycle of IH-2 were 21%→12% FiO_2_ for 90 s, 12% FiO_2_ for 15 s, and 12%→21% FiO_2_ for 75 s (20 cycles/h). **(B)** Changes in tumor volumes and body weights. **(C)** Comparison of tumor sizes from mice subjected to N, IH-1, or IH-2 conditions. Tumors were excised from mice on day 22 post-tumor injection (n = 9 for N; n = 7 for IH-1, and n = 9 for IH-2). **(D)** Comparison of tumor weights in the N, IH-1, and IH-2 groups. Data are presented as the mean ± S.E.M. ^*^P < 0.05 compared to N; ^†^*P* < 0.05 compared to IH-1; ^‡^*P* < 0.01 compared to IH-1; ^**^P < 0.01 compared to N and IH-1, as determined by the Kruskal–Wallis test, followed by the Mann–Whitney U test.

Next, we examined whether IH exposure after tumor xenografting could affect tumor growth. As depicted in Figure 4A, oxygen-saturation (SaO_2_) profiles in freely moving mice exposed to N, IH-1, or IH-2 conditions were obtained by mouse pulse oximetry. The SaO_2_ nadir under the IH-1 condition ranged from 67 to 73%. A slightly lower nadir of SaO_2_ was observed under the IH-2 condition (from 62 to 68%). At day 18 after injecting melanoma cells, the tumor volumes were higher in the IH-2 group than in the N and IH-1 groups (upper panel in Figure 4B). No differences in body weights were observed among the groups during the entire experimental period (lower panel in Figure 4B). Tumor weights were also significantly higher in the IH-2 group than in N and IH-1 groups (Figure 4C and 4D). Tumor volumes and weights did not differ between the N and IH-1 groups throughout the experimental period and at the time of sacrifice. One mouse each in the N and IH-2 groups and 3 mice in IH-1 group died during the experimental period, possibly due to tumor-induced death, which occurs frequently in B16F10-bearing mice [20]. We checked whether the loss of mice affected the final results regarding the tumor size and found that the mouse tumor volumes at the time of death were similar with the corresponding group’s average tumor volume (data not shown). Thus, our results were likely not affected by the reduction in data due to the lost mice.

### Increases in HIF-1α-dependent adaptive responses to hypoxia were frequency-dependent

**Figure 5:**
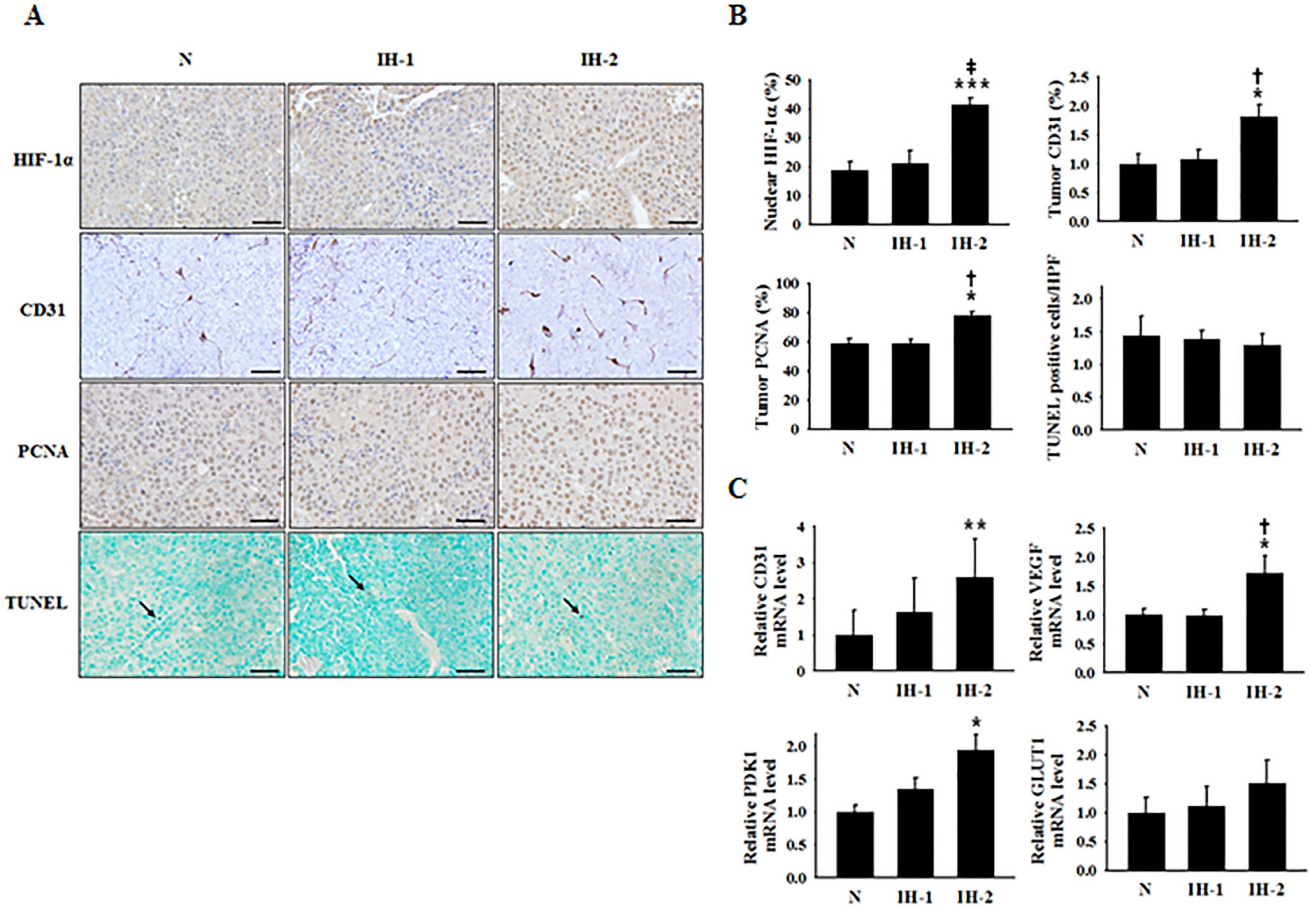
Expression of a cell-proliferation marker, endothelial cell markers, and hypoxia-response transcription factor in tumor tissues from mice subjected to normoxia (N), intermittent hypoxia-1 (IH-1), or intermittent hypoxia-2 (IH-2) conditions **(A)** Representative immunohistochemical staining images for HIF-1α, PCNA, CD31, and TUNEL-positive nuclei. Tumor tissues were collected on day 22 post-tumor inoculation. **(B)** Quantification of the % cells staining positive for nuclear HIF-1α, the % CD31-stained area, the % PCNA-positive cells, and the average number of TUNEL-positive cells/high-power field in the N, IH-1, and IH-2 groups. **(C)** Relative *CD31*, *VEGFA*, *PDK1, and GLUT1* mRNA-expression levels in tumors from mice in the N, IH-1, and IH-2 groups. Scale bars, 50 μm except for CD31 (100 μm). The arrow indicates TUNEL-positive nuclei.^*^P < 0.05 compared to the N group; ^**^P < 0.01 compared to the N group; ^***^P < 0.001 compared to the N group; ^†^*P* < 0.05 compared to the IH-1 group; ^‡^*P* < 0.01 compared to the IH-1 group, as determined by the Kruskal–Wallis test, followed by the Mann–Whitney U test.

IHC was performed to examine the expression levels of HIF-1α, CD31, and PCNA, as well as the number of TUNEL-positive nuclei in tumors from mice subjected to N, IH-1, or IH-2 conditions. Tumors in the IH-2 group showed significantly increased expression of those proteins versus the N and IH-1 groups, although the number of TUNEL-positive cells did not differ (Figure 5A and 5B). qRT-PCR analysis showed more increased expression of HIF-1α downstream target genes and the endothelial cell marker CD31 in tumors of the IH-2 group than in those of the N and IH-1 groups (Figure 5C), which confirmed the increased transcriptional activity of HIF-1α in the IH-2 group. However, we did not observe a significant difference between the mRNA level of HIF-1α itself in tumors of the IH-2 group, compared with the other two groups (Supplementary Figure 1C), implying that HIF-1α up-regulation in the IH-2 group was possibly due to increased protein stability, as opposed to elevated protein synthesis.

## DISCUSSION

In this study, we found that IH increased *in vivo* tumor growth, both with tumor cells that were preconditioned with IH and exposed to IH following tumor injection. Accelerated tumor growth was also associated with HIF-1-dependent hypoxia adaptation.

Hypoxia occurs commonly in solid tumors as the tumors proliferate more than the existing vascular can supply. Thus, adaptive responses to hypoxia (i.e., elevation of oxygen supply or compensation for the loss of energy) are an essential process for tumor growth and survival. HIF-1 is the best-known mediator of adaptive cellular responses to hypoxia and consists of a heterodimer of HIF-1α and HIF-1β, which are basic helix-loop-helix PAS proteins [21]. HIF-1 is hydroxylated by prolyl hydroxylases (PHDs) and degraded through the ubiquitin-mediated proteasome pathway under normoxia. Under hypoxia, PHDs are inactivated and HIF-1α is stabilized in the cytoplasm. Stabilized HIF-1α undergoes nuclear translocation and dimerizes with HIF-1β [22]. The HIF-1α/β dimer subsequently binds to hypoxia-response elements and modulates the transcription of several target genes, which are favor cancer progression (e.g., angiogenesis, glucose metabolism, cell proliferation, apoptosis, invasion, and metastasis). We found that HIF-1α and its downstream targets were up-regulated in tumors from mice subjected to 2 different IH protocols. These findings implied that a series of HIF-1-dependent, hypoxia-adaptation processes may play important roles in cancer progression promoted by IH. In a previous study that investigated the effect of IH on tumor growth in xenografted mice, circulating VEGF levels in the blood positively correlated with tumor weights [16]. However, no significant difference was observed between tumor VEGF and CD31 expression in mice subjected to IH. HIF-1α expression levels in tumors were also not examined. Another study provided evidence of increased VEGF expression and angiogenesis in human melanoma-derived tumors in mice, as well as elevated *in vitro* VEGF secretion in the same tumor cell lines when exposed to cyclic hypoxia [23]. Although the causal role of VEGF, which is elevated by IH, on tumor growth was not elucidated in those studies, it is clear that the upregulation of HIF-1-dependent VEGF expression promotes angiogenesis and contributes to tumor growth [24, 25].

While previous findings have shown that IH promotes cancer cell proliferation, as demonstrated by cell growth assays and cell cycle analysis [26], we did not observe any significant difference in biological behavior such as cell cycle progression or proliferation of melanoma cells that were maintained under normoxia or pre-IH-conditioned. These discrepancies might be due to differences in IH protocols (e.g., frequency and duration), the level of FiO_2_ used for hypoxia, cell line differences, and co-culture with other immune cells.

We also observed different findings between *in vitro* and *in vivo* experiments regarding HIF-1α-dependent hypoxic adaptation and cell-proliferative potential. The underlying mechanism by which hypoxia-adaptive responses and tumor growth in the Pre-IH group were more accelerated *in vivo* than were those in the N group is unknown. We speculate that long-term exposure to IH can make cancer cells acquire a persistent transformation, which is advantageous for adapting or proliferating under *in vivo* hypoxic environment, possibly through feed-forward regulation of biochemical/metabolic pathways or epigenetic modification [27]. This speculation is based partially on the findings that cancer cells pre-exposed to IH showed up-regulated expression of PDK1, revealing that those pre-trained cells were relatively prepared to adapt to IH conditions. This finding implies that when the xenografted cells formed a mass with intermittent hypoxia inside, those cells may overcome IH more easily. Previous results showed that an 8-week intermittent induction of HIF-1α, whose expression was regulated by tetracycline, promoted the growth or invasion of human glioma cells in mouse brains [27]. In addition, similar to our findings, the long-term intermittent induction of HIF-1α resulted in lost HIF-1α expression, but did not influence cell proliferation *in vitro*. These results cannot be directly compared to our results because HIF-1α expression was artificially driven (i.e., not by hypoxia), and the cell lines used differed from ours. Nevertheless, the previous data supports our findings that conditions that intermittently increase HIF-1α, such as IH, have long-lasting effects on subsequent malignant tumor progression *in vivo*.

Interestingly, tumor growth can depend on the frequency of IH; an epidemiological study revealed that only moderate-to-severe OSA (respiratory disturbance index ≥ 15) is independently associated with an increased risk for cancer and mortality [28]. Our data and previous epidemiological findings imply the existence of a threshold of IH frequency, beyond which malignance occurs more often. However, conflicting results regarding the relationship between the severity of OSA and cancer risk also exist. In a large cohort study conducted to analyze data from 10,149 patients who underwent a sleep study, the severity of OSA was not significantly associated with either the prevalence or incidence of cancer after controlling for possible confounding factors [29].

Factors that can determine the threshold of the malignant phenotype of cancer cells based on IH are unknown, but reactive oxygen species (ROS) are potential candidate molecules linking these processes. ROS up-regulates HIF-1α expression by stimulating synthesis or increasing its stability through Ca^2+^-dependent signaling pathways, including those involving phospholipase Cγ or protein kinase C and mTOR [30, 31]. In humans, OSA severity positively correlates with oxidative stress parameters [32, 33]. Therefore, ROS elevation above a threshold level may increase the stability of HIF-1α, thereby augmenting HIF-1-dependent adaptation responses to hypoxia, and contribute to tumor progression under IH-2 conditions. This possibility can be examined in further studies using ROS scavengers or conditional HIF-1α-knockout models.

The alteration of sympathetic outflow might also be a contributing factor for the different malignant phenotypes elicited by these IH protocols. Indeed, data from many epidemiologic and experimental studies have revealed that the β-adrenergic receptor signaling pathway was involved in the proliferation, metastasis, and angiogenesis of several types of tumors [34–36]. Accumulating evidence has indicated that IH is a powerful sympathetic activator [37, 38]. IH elicits both catecholamine synthesis and release *via* ROS-dependent tyrosine hydroxylase activation [39, 40]. It appears that HIF-1α mediates catecholamine-induced tumor malignancy in some types of cancer. Treatment with norepinephrine (NE), a ligand of the adrenergic receptor, up-regulated VEGF expression and angiogenesis in an HIF-1α-dependent manner in human breast, prostate, and hepatocellular cancer cells. However, pretreatment with either α- or β-adrenergic receptor blockers abolished such induction by NE [41]. Although we did not investigate sympathetic activity at the local or systemic level with the IH protocols designed for the present study, the possibility that catecholamine-associated adrenergic receptor signaling determines malignant phenotypes should be investigated in further studies.

In addition, the increased genomic instability and activation of cancer stem cells (CSCs) might be regarded as possible factors linking IH with enhanced cancer progression [42]. Those are likely to be caused by impaired activities of enzymes responsible for DNA metabolism [43] and HIF-mediated expression of CSCs factor such as Oct4, c-Myc, Wnt, and Notch [44].

In summary, we observed that both pre-IH conditioning and IH exposure in tumor-bearing mice promoted tumor growth *in vivo*, and the accelerated progression was associated with HIF-1-dependent adaptive responses to hypoxia. HIF-1-dependent angiogenesis promotes tumor growth, survival, or metastasis, antiangiogenic therapy, and targeting up-regulated HIF-1 expression by IH could be an attractive therapeutic modality [45]. Further mechanistic studies involving pharmacologic inhibition or genetic manipulation are required to elucidate the exact mechanism by which IH promotes tumor progression.

## MATERIALS AND METHODS

### Cell culture and *in vitro* preconditioning by IH

Mouse melanoma (B16F10) cells were obtained from Korean Cell Line Bank (Seoul, Korea) and were cultured in 100-mm dishes in Dulbecco’s modified Eagle’s medium containing 10% fetal bovine serum, 100 U/ml penicillin, and 100 μg/ml streptomycin. Prior to preconditioning, the cells were maintained in a humidified incubator at 37°C and 5% CO_2_. For Pre-IH experiments, cells were exposed to 12 consecutive cycles of hypoxia for 50 min (1% O_2_, 5% CO_2_, 37°C; balanced N_2_ and water vapor) followed by 10 min of normoxia (N conditions; 21% O_2_, 5% CO_2_, 37°C; balanced N_2_ and water vapor) during a day. After completing the 12 cycles, the cells returned to the N conditions and maintained for 12 h. This procedure was repeated for 7 consecutive days. During this period, the cells were sub-cultured twice after being returned to the N conditions. N-conditioned cells were continuously maintained in the normoxic chamber during the same period. After 7 days of Pre-IH or N conditioning, 2 × 10^5^ B16F10 cells in 100 μl of phosphate-buffered saline (PBS) were subcutaneously injected into the backs of mice treated with no hypoxic stimulus. Tumor growth was assessed by externally measuring tumor volumes beginning on day 10 at 2–3 day intervals after the injection of cancer cells. The tumor volume (V) was estimated by externally measuring its length (L) and width (W) with a caliper (V = W^2^ × L/2). On day 19, the mice were sacrificed and the tumors were excised. The excised tumors were rinsed in cold PBS and processed for histological examinations and gene-expression studies.

### *In vivo* IH model

Thirty male, 8-week-old C57BL/6 mice (Orient Bio, Seongnam, Korea) were used in this study. All animals were maintained in a temperature-controlled room with alternating 12-h cycles of light and dark (light on at 8:00 a.m.). All experimental procedures were approved by The Institutional Animal Care and Use Committee of the Seoul National University College of Medicine (SNU-150213-1). After subcutaneous injection of the B16F10 cells, the animals were randomly divided into 3 groups: 1) N (n = 10), 2) IH-1 (n = 10), and 3) IH-2 (n = 10). IH was applied to the mice on the day after tumor cell injection using an OxyCycler A84XOV gas controller (BioSpherix, NY, USA). The time course of oxygen-saturation profiles in mice was monitored using a MouseOx Plus oximeter (Starr Life Sciences, PA, USA). Mice in the IH-1 group were subjected to IH (90 s of 12% FiO_2_ followed by 270s of 21% FiO_2_, [10 cycles/h]) for 8 h/day during the light period (09:00–17:00) for 21 consecutive days. Mice in the IH-2 group were subjected to IH with alternating cycles of 90s (12% FiO_2_ followed by 90 s of 21% FiO_2_, 20 cycles/h) for the same duration as mice in the IH-1 group. Mice in the N group were maintained in a chamber supplied with continuously circulating room air. Mice were subcutaneously injected with 1 × 10^6^ B16F10 cells suspended in 100 μl of PBS on the back. Tumor growth was assessed beginning on day 8 at 2–3 day intervals after tumor cell injection, using the same method in the preconditioning experiments. On day 22, the mice were sacrificed and the tumors were excised and weighed. The excised tumors were processed as performed in the preconditioning experiment.

### Cell cycle analysis and BrdU-based cell proliferation assay

Cell cycle analysis was performed in normoxic or preconditioned B16F10 cells for 7 days. Before treatment for cell cycle analysis, preconditioned or non-conditioned B16F10 cells were incubated under normoxia at 37°C overnight. Cells were then harvested, washed with PBS, and fixed in cold 75% ethanol overnight at -20°C. After washing and re-suspension in PBS, the fixed cells were treated with RNase A (Sigma-Aldrich) for 40 min at 37°C. Propidium iodide was then added to each sample. Cell proliferation was determined using the FITC BrdU Kit (Becton Dickinson [BD], USA), according to the manufacturer’s instructions, at the same time point used for cell cycle analysis. Briefly, 1 × 10^6^ B16F10 cells grown in 60-mm dishes were treated with BrdU-incorporation solution and incubated for 1 h at 37°C, followed by fixation and permeabilization. After DNase treatment to expose the incorporated BrdU for 1 h at 37°C, the fixed cells were incubated with an anti-BrdU antibody for 20 min at room temperature. Total DNA levels were determined by 7-AAD staining. BrdU-incorporated or 7-AAD stained cells were analyzed on a BD FACSCanto^TM^ flow cytometer (BD Bioscience, USA), and DNA histograms were constructed using BD FACSDiva software, version 8.0.1.

### Immunohistochemical staining and TUNEL assay

For immunohistochemical staining, tumors excised from mice were fixed in 4% paraformaldehyde at 4 for 24 and embedded in paraffin. For antigen retrieval, 4-μm thick sections were autoclaved at 121°C for 10 min in 100 mmol/L citrate buffer (pH 6.0; Dako, Glostrup, Denmark). After treatment with 3% hydrogen peroxide in methanol for 10 min in a moisture chamber, the sections were incubated with 2% blocking serum for 1 h at room temperature and then incubated overnight with a primary anti-HIF-1α (1: 100; Novus Biologicals, CO, USA), anti-CD31 (1: 200; Thermo Scientific, USA), or anti-PCNA (1: 1000; Santa Cruz Biotechnology, Inc., USA) antibody at 4°C. After overnight incubation with the primary antibodies, the sections were incubated for 1 h with a secondary antibody provided in GBI Polink HRP Kit (Golden Bridge International, Inc., USA). Then, the sections were developed using 3,3′-diaminobenzidine. Hematoxylin was employed for counter staining (Vector Laboratories, Inc., CA, USA). Sections were analyzed with a bright-field microscope (BX-51; Olympus, Tokyo, Japan) equipped with a DP70 camera (Olympus). To evaluate the results of IHC staining, 5 random high-power fields per slide (HPF; X 400) with no necrotic areas were acquired from all mice (10 tumors in the N group and 10 tumors in the Pre-IH group; or 9 tumors in the N group, 7 tumors in the IH-1 group, and 9 tumors in the IH-2 group), and the percentages of cells showing positive nuclear HIF-1α or PCNA expression, and the %CD31-positive areas were determined using the Image J program. IHC images were analyzed under blind conditions. These levels are presented as the mean values of the images. To determine the degree of apoptosis, we performed TUNEL assay using the APO-BRDU-IHC Kit (Novus Biologicals, CO, USA), according to the manufacturer’s protocols. The extent of apoptosis was expressed as the mean number of TUNEL-positive nuclei per HPF.

### Western blot analysis

Cells were rinsed in ice-cold PBS and harvested in lysis buffer. Equivalent amounts of protein were separated by 8% SDS-PAGE and transferred to a polyvinylidene membrane (Millipore, MA, USA). Membranes were blocked with 5% nonfat dried milk for 1 h at room temperature; incubated at 4°C overnight with either a rabbit polyclonal anti-HIF-1α antibody (1: 1000; customized), a rabbit polyclonal anti-HIF-2α antibody (1: 1000; Cell Signaling Technology, MA, USA), or a rabbit polyclonal anti-PDK1 antibody (1: 1000; Enzo Life Sciences Inc., NY, USA); and then incubated for 1 h with a horseradish peroxidase-conjugated secondary antibody (1: 5000). Signals were detected using the Luminata^TM^ Crescendo Western HRP Substrate solution (Millipore, MA, USA). β-tubulin (1: 5000; Santa Cruz Biotechnology, Inc.) was detected as a loading control.

### RNA isolation and qRT-PCR

Total RNA from excised tumor tissue was extracted using the TRIzol reagent (Ambion). One microgram of total RNA was reverse transcribed into complementary DNA using the Tetro cDNA Synthesis Kit (Bioline, London, UK), according to the manufacturer’s instructions. qRT-PCR was performed using an iQ5 Real-Time PCR System (Bio-Rad Laboratories, Hercules, CA, USA). All reactions were performed in duplicate, and the specificity was examined by melting-curve analysis. Target-specific primers with the following sequences were used for amplification: *HIF1A*, F: 5′-CTGCAGGGTGAAGAATTACT-3′ and R: 5′-GTTTGTGCAGTATTGTAGCC-3′; *VEGFA*, F: 5′-TGC AGGCTGCTGTAACGATG-3′ and R: 5′-CCTCGG CTTGTCACATTTTTCT-3′; *PECAM1 (CD31)*, F: 5′-TC ACCATCAACAGCATCCAT-3′ and R: 5′-GGTGCTGA GACCTGCTTTTC-3′; *PDK1*, F: 5′-CAACTACCCTTGG TATGGTATGGG-3′ and R: 5′-CGTGGGAGATAAG AAGACCATCTG-3′; *GLUT1*, F: 5′-AACATGGAACC ACCGCTACG-3′ and R: 5′-GTGGTGAGTGTGGTG GATGG-3′; *ACTB*, F: 5′-GGCTGTATTCCCCTCC ATCG-3′ and R: 5′-CCAGTTGGTAACAATGCCATGT-3′. The 2-delta-delta comparative cycle threshold (Ct) method was used to compare the relative gene-expression levels, which were normalized against β-actin mRNA expression.

### Statistical analysis

All data were expressed as the mean ± standard error of the mean (S.E.M). Differences in the means were evaluated using the non-parametric Kruskal–Wallis test. Mann–Whitney *U*-tests were used for the post-hoc analyses and comparisons of variables between 2 independent groups. All statistical analyses were performed using IBM SPSS, version 20.0 (SPSS; Chicago, IL, USA), and *P* < 0.05 was considered to indicate statistical significance.

## SUPPLEMENTARY MATERIALS FIGURE


