# Supplementary material for: Accelerated tumor growth under intermittent hypoxia is associated with hypoxia-inducible factor-1-dependent adaptive responses to hypoxia
Source: Oncotarget. 2017 Jun 27;8(37):61592–603. doi: 10.18632/oncotarget.18644 (PMC5617448; doi:10.18632/oncotarget.18644)
Supplement: Supplementary file 1 [file oncotarget-08-61592-s001.pdf]

# Accelerated tumor growth under intermittent hypoxia is associated with hypoxia-inducible factor-1-dependent adaptive responses to hypoxia

## SUPPLEMENTARY FIGURE

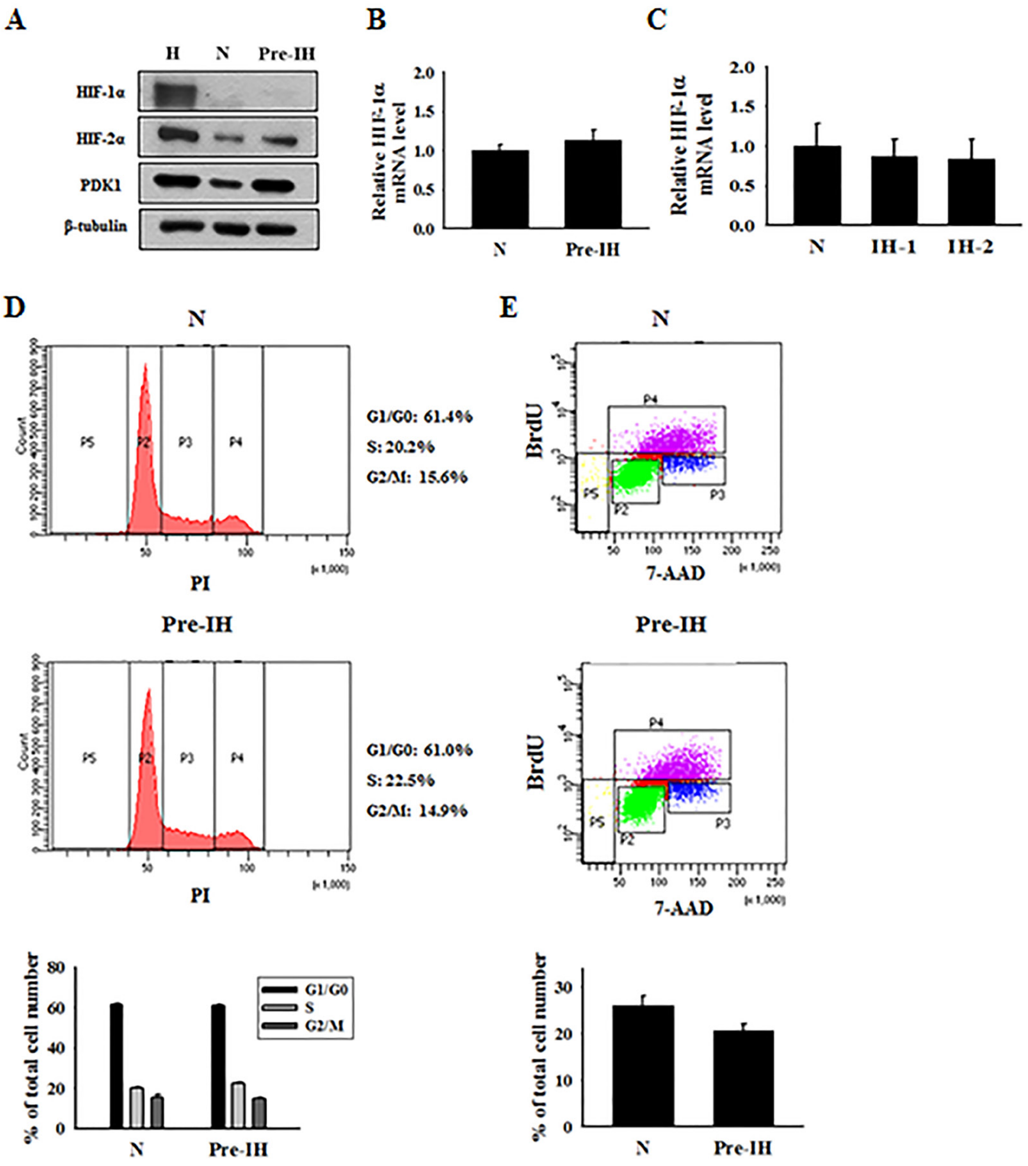


**Supplementary Figure 1: Characteristics of pre-intermittent hypoxia (Pre-IH) conditioned B16F10 cells before inoculation into mice and relative *HIF1A* mRNA expression in tumors from mice in the N or Pre-IH groups and N, IH-1, and IH-2 groups. (A)** A representative western blot image showing HIF-1α, HIF-2α, and PDK1 expression in extracts from cells subjected to 8 h of continuous hypoxia (H; 1% $O_2$) and 7 days (84 cycles) of no conditioning (N), or Pre-IH. **(B)** Comparison of relative *HIF1A* mRNA expression levels in tumors from mice in the N or Pre-IH groups. **(C)** Comparisons of relative *HIF1A* mRNA expression in tumors from mice in the N, IH-1, and IH-2 groups. **(D)** Cell cycle analysis of B16F10 melanoma cells subjected to 7 days of N or Pre-IH by flow cytometry, using propidium iodide (n = 3 for each group). The graph represents the % of cells in each cell cycle stage. **(E)** BrdU-based cell-proliferation assay of B16F10 melanoma cells subjected to 7 days of N or Pre-IH (n = 3 for each group). The graph represents the % of BrdU-incorporating cells. The data are presented as the mean ± S.E.M. Statistical significance was determined by the Mann–Whitney U test.
